# The Neural Correlates of the Interaction between Semantic and Phonological Processing for Chinese Character Reading

**DOI:** 10.3389/fpsyg.2016.00947

**Published:** 2016-06-23

**Authors:** Xiaojuan Wang, Rong Zhao, Jason D. Zevin, Jianfeng Yang

**Affiliations:** ^1^Key Lab for Behavior & Cognitive Neuroscience of Shaanxi Province, School of Psychology, Shaanxi Normal UniversityXi’an, China; ^2^Department of Psychology and Linguistics, University of Southern California, Los AngelesCA, USA

**Keywords:** visual word reading, fMRI, stimulus correlation approach, Chinese, connectionist model

## Abstract

Visual word recognition involves mappings among orthographic, phonological, and semantic codes. In alphabetic languages, it is hard to disentangle the effects of these codes, because orthographically well-formed words are typically pronounceable, confounding orthographic and phonological processes, and orthographic cues to meaning are rare, and where they occur are morphological, confounding orthographic and semantic processes. In Chinese character recognition, it is possible to explore orthography to phonology (O-P) and orthography to semantics (O-S) processes independently by taking advantage of the distinct phonetic and semantic components in Chinese phonograms. We analyzed data from an fMRI experiment using lexical decision for Chinese characters to explore the sensitivity of areas associated with character recognition to orthographic, phonological, and semantic processing. First, a correlation approach was used to identify regions associated with reaction time, frequency, consistency and visual complexity. Then, these ROIs were examined for their responses to stimuli with different types of information available. These results revealed two neural pathways, one for O-S processing relying on left middle temporal gyrus and angular gyrus, and the other for O-P processing relying on inferior frontal gyrus and insula. The two neural routes form a shared neural network both for real and pseudo-characters, and their cooperative division of labor reflects the neural basis for processing different types of characters. Results are broadly consistent with findings from alphabetic languages, as predicted by reading models that assume the same general architecture for logographic and alphabetic scripts.

## Introduction

Visual word recognition involves mappings among orthographic, phonological, and semantic codes. Reading aloud is thought as the cooperative division of labor between orthography-to-phonology and orthography-to-semantics processes (Harm and Seidenberg, 2004; Carreiras et al., 2014). This view has received the support of a considerable amount of evidence from studies of behavior (Strain et al., 1995), neuropsychology (Woollams et al., 2007) and computational modeling (Harm and Seidenberg, 2004), as well as a small amount of neural evidence from alphabetic languages (Frost et al., 2005; Graves et al., 2010; Boukrina and Graves, 2013). In alphabetic writing systems, orthography (O) and phonology (P) are highly confounded, O-P mappings are overwhelmingly regular, and so it’s hard to know whether these interactions are due to the functioning of the neural networks involved in reading, or due to unavoidable correlations in stimulus materials. Nonetheless, a number of recent studies have suggested that the functional organization of the reading system in the brain is generally similar across alphabetic and logographic writing systems (Nakamura et al., 2012; Rueckl et al., 2015; Wang et al., 2015).

In the literature of fMRI studies of word reading from alphabetic languages, a small amount of neural evidence has showed the interaction between the neural routes of orthography-to-phonology (O-P) and orthography-to-semantics (O-S) processing. For example, Frost et al. (2005) found that highly imageable words reduced the activation in left inferior frontal gyrus (IFG) for O-P processing, and correspondingly increased the activation at left middle temporal gyrus (MTG) and angular gyrus (AG) for semantic processing. The result indicates a trade-off between phonology and semantic processing in word reading. In a recent study, Boukrina and Graves (2013) used an effective connectivity algorithm to show that areas supporting semantic processing [e.g., inferior temporal sulcus (ITS)] interacted with phonological areas [e.g., posterior superior temporal gyrus (pSTG)]. The connectivity from ITS to pSTG changed as a function of word properties, in which the connectivity emerged for high- compared to low-imageability words, and for low-consistency words under certain conditions.

The neural basis of O-P processing is consistent in many studies of alphabetic languages. It relies on connections from the ventral occipitotemporal (vOT) region to the temporoparietal junction (Pugh et al., 2000; Price, 2012). In a meta-analysis of 35 neuroimaging studies (Jobard et al., 2003), grapheme–phoneme conversion was proposed to rely on left pSTG, supramarginal gyrus, and the opercular part of IFG. This neural route is in agreement with the findings of the most recent meta-analysis (Cattinelli et al., 2013; Taylor et al., 2013) and the literature review (Carreiras et al., 2014).

On the contrary, the neural basis of O-S processing is far from clear. Price (2012) reviewed the literature and suggested an O-S route as the connection from inferior occipital (iO) to MTG via vOT. This route is in agreement with previous meta-analysis (Jobard et al., 2003). However, it is not consistent with the finding from a work of the effective connectivity (Richardson et al., 2011). Using dynamic causal modeling, Richardson et al. (2011) tested the sub-routes of the connection from the visual cortex to the left temporal lobe. They suggested that O-S processing relies on the link from iO to anterior superior temporal sulcus (STS) via vOT.

The lack of the consensus of the neural basis of O-S processing may be because it is hard to investigate in alphabetic word reading. Alphabetic writing systems represent the phonological structure of the language more or less directly and componentially. Pronunciations can be computed directly from spelling, resulting in a relatively limited role for semantics in reading aloud (Raman and Baluch, 2001). Thus, unlike the consistent findings of the neural basis of O-P processing, the cortical substrate of semantic processing consists of extending left-brain areas in anterior temporal lobule (ATL), MTG, AG, and IFG (see reviews, Binder et al., 2009; Carreiras et al., 2014).

Considering the difficulty of testing the neural basis of O-S processing, prior studies mainly examined the neural correlates of lexico-semantic processing. To do this, researchers commonly manipulated the stimulus property and the task demand. Typically, semantic tasks (e.g., meaning relatedness judgment) recruited more activation at semantic regions than a phonological task (e.g., rhyming judgment, Booth et al., 2006; Vigneau et al., 2006). Stimulus properties are also manipulated to examine additional lexico-semantic processing for particular types of word. For example, according to the connectionist approach, if both the frequency and spelling–sound consistency is low, word reading requires additional input via a semantically mediated pathway (Plaut et al., 1996; Harm and Seidenberg, 2004). Consequently, low frequency irregular/inconsistent words recruited more activation at left AG and MTG for lexico-semantic processing (Taylor et al., 2013). Imageability is another stimuli manipulation on the semantic attributes of the whole word. High imageable word facilitated recognition, particularly for words that depend on semantics for correct naming (Strain et al., 1995), and recruited more activation at left MTG and AG for semantic processing than low imageability words (Frost et al., 2005). These studies suggest a neural pathway for semantic processing contributed to the word reading, and its interaction with the neural pathway for phonological processing. But research on alphabetic writing systems cannot disambiguate lexico-semantic from orthography-to-semantic processing.

The O-S processing can be test in Chinese character reading since the unique logographic properties of the Chinese writing system. In modern Chinese, more than 80% characters are phonograms (Zhu, 1988). Phonograms consist of two components, a phonetic indicating the character’s pronunciation, and a semantic radical indicating the character’s semantic category. The pronunciation of the phonetic influenced Chinese character reading by showing the regularity and consistency effect (Hue, 1992; Lee et al., 2005; Yang et al., 2009). The meaning of some phonetics, as well as the semantic component, was also found to influence processing of Chinese characters (Feldman and Siok, 1999; Zhou and Marslen-Wilson, 1999). Thus, semantic processing has been proposed to interact with phonological processing in the studies of patients (Han et al., 2005; Bi et al., 2007), children (Shu et al., 2005), and computer modeling (Yang et al., 2013).

However, only a few neuroimaging studies in Chinese have addressed the neural basis of O-S processing and its interaction with O-P processing. Based on the task manipulation, the semantic processing in Chinese reading relied on the left MTG and anterior ventral IFG (Wu et al., 2012). The left MTG associated with meaning association judgment than rhyme judgment (Booth et al., 2006), and anterior ventral IFG was more active for meaning judgment than a perceptual control task (Chou et al., 2009). To our knowledge, no existing fMRI study has examined the cortical substrate for processing of semantic cues in the Chinese orthography. Furthermore, the neural network of character reading is more uncertain when considering the language differences. Although, pSTG was consistently active for O-P processing in alphabetic word reading (Paulesu et al., 2000), it was null or deactivated for Chinese character either in naming (Tan et al., 2001a) or lexical decision (LD) task (Yang et al., 2012), whereas, it was active in Chinese during continuous reading (Wang et al., 2015) and semantic judgment tasks (Rueckl et al., 2015). That is, the task demands might influence the previous findings of the Chinese reading network.

In the current study, we reanalyze the data from an fMRI study of Chinese character reading (Yang et al., 2012) to investigate the independent contributions of orthographic, phonological, and semantic processing. Rather than examining semantic or phonological processing manipulated via instructions, we can show it in the context of a single task. To do this, we first used a stimulus correlation approach (Graves et al., 2010) to identify reading network overlaps with alphabetic languages in correlation with reaction time (RT), word frequency, visual complex, and orthography-to-phonology consistency. The parametric approach can use one data set to reveal distinct brain areas those are correlated with highly intercorrelated variables (Hauk et al., 2008). Then, ROIs were examined for their responses to stimuli with different types of information available. In this way, we can investigate the relative contribution of the orthographic, phonological, and semantic processing on character reading. Based on data from alphabetic languages and task manipulations in Chinese fMRI studies, we predicted that the orthographic processing mainly relied on the visual cortex, and orthography-to-semantics processing would rely on the activation of posterior MTG, and inferior parietal cortex (mainly located at the angular and the supramarginal gyrus). The orthography-to-phonology processing would rely on IFG, but not on the posterior STS due to the absence of grapheme-to-phoneme corresponds for Chinese characters. The findings will shed a light on the general architecture for reading of logographic and alphabetic scripts.

## Materials and Methods

Data from 16 participants (18–25 years) that participated in a previously published study (Yang et al., 2012) were reanalyzed in the current study. Written informed consent was obtained from each participant, and the study was approved by the Institutional Review Board of the State Key Laboratory of Cognitive Neuroscience and Learning at Beijing Normal University. A complete description of the methods is described in the initial publication. Here, we provide a concise description of the aspects of the data most relevant for the new analyses. There is no overlap between the goal of the present study, which focused on the interaction among brain regions in LD task, and the prior study, which characterized activation of brain regions driven by stimulus properties and tasks demands.

### Materials

Sixteen participants performed LD task on six types of word-like stimuli: real characters (RW), pseudo-characters containing Phonology plus Semantics (PS), Orthography plus Semantics (OS) or Only Orthography (OO), and two types of orthotactic violations: the reversed radicals (RR) condition, created by reversing the position of components in the OO pseudo-characters, and nonsense strokes (NN) condition, created by randomizing the individual strokes in RR stimuli. Ninety additional real characters (Filler) were included to balance the number of “word” responses in LD task. Here, we analyzed the real characters both including 30 RW and 90 fillers.

By comparing the neural difference between two types of pseudo-character, we can reveal three processing pathways: PS > OS for phonological processing, OS > OO for semantic processing, and OO > RR for orthographic processing. If a region is active both for PS > OS and for OO > RR, its function may be related to orthography-to-phonology mapping. In the same way, the neural basis of orthography-to-semantics mapping can be identified by combining the activity for OS > OO and OO > RR.

### Procedure

Participants were familiarized with the LD task and symbol detection (SD) task, then lay comfortably in the scanner and viewed stimuli via rear projection during the tasks. Participants performed SD task first. Both tasks were run using fast random interval event-related designs. On each trial, a 200 ms fixation cross was presented, followed by a stimulus presented for 500 ms, followed by a randomly jittered inter-trial interval (mean of 5.3 s, range from 1 to 14 s). Stimulus presentation was controlled, and response time and accuracy were recorded using E-Prime software.

In the LD task, participants were asked to respond by button press with their right index finger to real characters, and with their middle finger to all non-character stimuli. This task was completed in two consecutive runs of 135 trials (15 trials for each of the six critical conditions and 45 filler trials). The data of SD task was not included in this study.

### MRI Acquisition

Functional and anatomical images were collected using a 3T Siemens Magnetom Trio Timsyngo MR system, with a 12-channel head coil in the State Key Laboratory of Cognitive Neuroscience and Learning of Beijing Normal University. Functional images were collected using a gradient-recalled-echo echo-planar imaging (EPI) sequence sensitive to the BOLD signal. Forty-one axial slices were collected with the following parameters: TR = 2500 ms, TE = 30 ms, flip angle = 90°, FOV = 20 cm, matrix = 64 × 64, 3 mm thickness, yielding a voxel size of 3.125 mm × 3.125 mm × 3 mm, interleaved slices with no gap. The LD task was accomplished in two runs of 332 volumes (13 m, 50 s) including four TRs of rest at the beginning and end of each run.

Following the acquisition of functional data, high resolution T1-weighted anatomical reference images were obtained using a 3D magnetization prepared rapid acquisition gradient echo (MPRAGE) sequence, TR = 2530 ms, TE = 3.45 ms, flip angle = 7°, FoV = 25.6 cm, matrix = 256 × 256 with 1 mm thick sagittal slices.

### Data Analysis

#### MRI Data Analysis

Functional data were analyzed using AFNI (Cox, 1996); program names appearing in parentheses below are part of the AFNI suite. Cortical surface models were created with FreeSurfer (available at: http://surfer.nmr.mgh.harvard.edu/), and functional data projected into anatomical surface space using SUMA (AFNI and SUMA are available at http://afni.nimh.nih.gov/afni, Saad et al., 2004; Argall et al., 2006).

#### Preprocessing

After reconstructing 3D AFNI datasets from 2D images (to3d), the anatomical and functional datasets for each participant were co-registered using positioning information from the scanner. The first three volumes were discarded, and functional datasets preprocessed to correct slice timing (3dTshift) and head movements (3dvolreg), reduce extreme values (3dDespike) and detrend linear and quadratic drifts (3dDetrend) from the time series of each run, with no smoothing or filtering.

#### General Linear Models and Contrasts

Preprocessed data for 2 runs of the LD task were analyzed in a general linear model (GLM) including six regressors of no interest (Six estimates of head movement from motion correction from 3dVolreg). The six experimental regressors were hypothetical hemodynamic response functions (HRFs) constructed by convolving the stimuli presenting time of each condition (Real, PS, OS, OO, RR, and NN) with a model HRF (waver). The contrasts were created by comparing real characters and combined pseudo-characters (PS, OR, OO, and RR) to NN condition.

#### Stimulus Correlation Analysis

To test for stimulus property-related activation, a real character-related time serial dataset was synthesized by selecting sub-brick and matrix column for real characters from initial GLM (3dSynthesize). Resulting time serial dataset was analyzed in separately GLMs including two regressors: one binary variable for onsets of trials and another continuous, mean-centered values of each stimulus property: RT in the LD task, orthography-to-phonology consistency degree, frequency and number of strokes. The frequency was from a corpus of Modern Chinese Frequency Dictionary (1986). The consistency degree was computed as the summed frequency of friends divided by the summed frequency of all family members (including the character itself, as in Shu et al., 2003). The correlation matrix of stimulus properties is given in **Table 1**. Frequency is significantly correlated to RT and strokes number, but not to consistency. There is no correlation among consistency, RT and strokes number.

**Table 1:** Correlations among stimuli properties.

	Frequency	Consistency	Strokes *n*	Reaction time
Frequency	1.00			
Consistency	-0.04	1.00		
Strokes n	-0.26^∗∗^	0.12	1.00	
Reaction Time	-0.39^∗∗∗^	-0.11	0.14	1.00

*^∗∗^*p* < 0.01; ^∗∗∗^*p* < 0.001.*

#### Group Analysis

Group analyses were conducted for each contrast or correlation by comparing the mean coefficients from all participants to zero for each node in the standard surface (3dttest). The resulting surface was mapped to an AFNI volume based on a mesh of averaged brain (3dSurf2Vol), resulting in maps with *t* statistics for each voxel for each contrast. The volume datasets were then converted to Talairach space (@auto_tlrc, using the N27 template) at 2 mm × 2 mm × 2 mm resolution. Activation maps and regions reported as active in tables were obtained by first thresholding individual voxels at *p* < 0.005 (uncorrected), and then applying a subsequent cluster-size threshold (at least 50 voxels) based on Monte Carlo simulation (AlphaSim), resulting in a corrected threshold of *p* < 0.05.

#### Surface Reconstruction and Projection of Functional Data into Surface Space

Surface-based spatial normalization of anatomical and functional data was accomplished using Freesurfer (Fischl et al., 1999) and SUMA (Argall et al., 2006). Anatomical data were reconstructed (to3d), and a surface model for each participant was made with Freesurfer: cortical meshes were extracted from the structural volumes, then inflated to a sphere and registered anatomically. Using the surface atlas, an averaged subject was created by averaging surfaces, curvatures, and volumes from all subjects. The averaged surface was converted into SUMA and was then put into a standard mesh on the SUMA surfaces. The standard mesh was then converted to a volume and transformed (using @auto_tlrc, to the N27 template) to Talairach space (Talairach and Tournoux, 1988) for visualization and reference purposes. Functional data were normalized by transforming volumes resulting from AFNI analyses into surface representations using the standardized surfaces, and computing averages over surfaces.

#### Region of Interest (ROI) Analysis

To test the function of specific regions sensitive to real characters’ properties, nine ROIs were identified in the left hemisphere by combining areas significantly correlated with parametric variables: six ROIs were highly correlated to frequency, including three ROIs with positive correlations, in temporoparietal junction, MTG and Inferior Parietal Lobule, and three ROIs with negative correlations in pre-central/middle frontal gyrus, insula, and fusiform gyrus; Two ROIs were highly correlated to number of strokes for positive region at inferior occipital gyrus and negative region at AG; One ROI was negatively correlated with consistency degree at IFG. In each ROI, the mean beta value was computed and compared across four types of pseudo-characters.

## Results

### Overlapping and Distinct Regions for Real and Pseudo-characters

**FIGURE 1:**
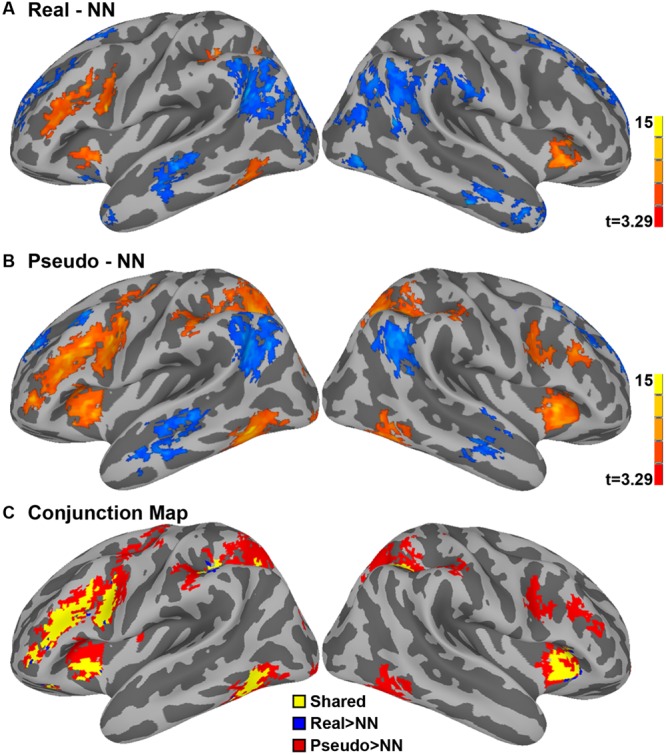
**The contrasts of Real > NN **(A)** and Pseudo > NN **(B)** showed a similar neural network for lexical decision (LD) task.** The conjunction map **(C)** showed pseudo-characters activated more regions at right hemisphere and more spatial extending regions at left hemisphere than real characters.

To identify the neural networks for reading Chinese characters, we first analyzed the GLM contrasts for both Real and Pseudo-character against the nonsense strokes (NN condition) baseline. We subsequently conducted a conjunction analysis to examine whether the neural networks were driven by stimulus type. The results were presented in brain maps (**Figure 1**), and the clusters were listed in **Table 2**.

**Table 2:** Brain regions activated more for real characters and pseudo-characters contrasted to stroke patterns (NN).

Region	H	BA	Volume	*t*	*x*	*y*	*z*
**Real > NN**							
Mid. frontal G.	L	9	260	7.78	-35	18	34
Inf. frontal G.	L	9	172	12.31	-46	4	22
Insula	L	13	153	7.80	-35	22	1
	R	13/45	182	8.89	31	28	2
Medal frontal G.	B	8	71	6.48	-1	25	51
Fusiform G.	L	37	134	7.99	-50	-54	-15
Precuneus	L	40	311	7.77	-29	-67	34
Inf. parietal L.	L	40	64	5.88	-54	-40	41
	L	40	50	7.40	-37	-46	43
**Pseudo > NN**							
Sup. parietal lobe	L	31	1376	17.85	-27	-68	27
	R	7	824	10.48	27	-66	31
	R	40	226	8.33	58	-30	40
Sup. frontal G.	B	6	726	10.47	-5	4	63
Mid. frontal G.	L	9	1056	12.18	-35	18	34
	L	4	83	6.14	-31	-10	50
	R	9	136	7.92	48	24	34
Precentral G.	L	4/3	77	6.51	-21	-20	58
	L	4	53	5.46	-37	-12	49
Inf. frontal G.	L	13	513	11.23	-33	22	3
	R	13	379	11.20	31	24	6
	R	6	240	7.43	37	1	35
Mid. occipital G.	L	19	105	7.09	-33	-88	15
Fusiform G.	L	37	466	13.28	-50	-56	-15
	L	18	111	6.63	-17	-95	-20
	L	20	95	5.57	-39	-18	-25
	R	37	233	9.48	50	-54	-20
Lingual G.	L	18	59	4.45	-21	-55	2
Cingulate G.	L	24	133	10.54	-5	-0	30
Posterior cingulate	R	19	57	5.54	15	-63	3
Culmen	L	19/18	67	4.92	-7	-59	-1
Cuneus	L	18	52	6.53	-15	-72	5

*H = left (L), right (R), or both (B) hemisphere; BA, Broadmann’s area; Volume is given in number of voxels (2 mm × 2 mm × 2 mm); Coordinates (*x, y, and z*) for the peak active voxel in each cluster are given with reference to the MNI atlas. For brain regions: Sup., superior; Mid., middle; Inf., inferior; G., gyrus.*

As viewed side-by-side, the contrasts of Real > NN (**Figure 1A**) and Pseudo > NN (**Figure 1B**) showed a similar neural network in the LD task. As shown in **Figure 1C**, the conjunction map revealed overlapping regions in bilateral intraparietal sulcus (IPS) and insula, and in left hemispheric areas including IFG/MFG, precentral gyrus and fusiform gyrus (FFG). The shared regions for two types of stimuli are largely consistent with the previous contrast of task vs. rest in numerous studies of Chinese character recognition (Peng et al., 2004; Liu et al., 2007; Wang et al., 2011) and meta-analysis (Bolger et al., 2005; Tan et al., 2005; Wu et al., 2012).

The distinct regions observed only for the contrast of Pseudo > NN, not for Real > NN, were mainly located in bilateral IPS, right FFG, right IFG, and right MFG. Also, a greater spatial extent of activation for pseudo-characters was observed in these shared regions.

### Brain Regions Sensitive to Stimulus Properties

**FIGURE 2:**
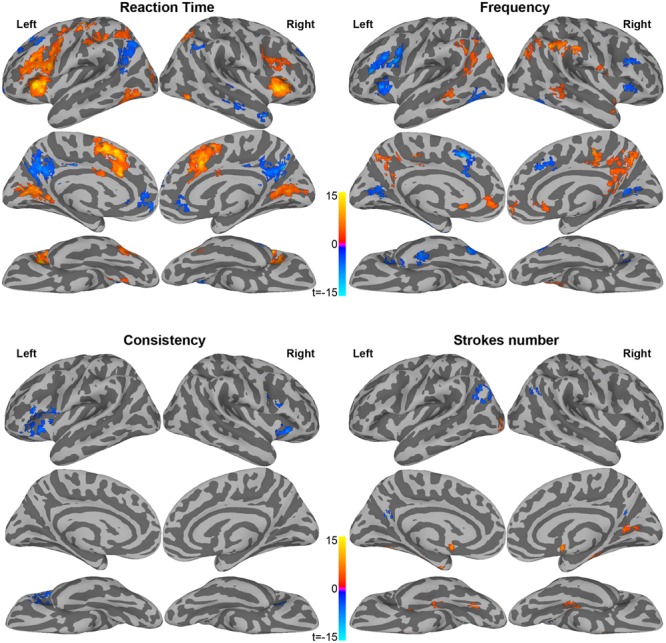
**Brain areas showing a significant correlation between neural activity and reaction time in LD task, frequency, consistency degree, and stroke numbers**.

Using a multi-parametric approach, we explored the brain regions sensitive to visual/orthographic, phonological, and lexical processing in reading Chinese characters. We characterized stimulus-related regions by computing the correlations with BOLD signal level for RT and stimulus properties of characters: character frequency, consistency degree, and the number of strokes. The multi-parametric approach was generally consistent with the networks of Chinese character reading identified in the contrasts described above. Maps of these correlations are shown in **Figure 2**.

Correlations between BOLD signal and RT were observed in the Chinese reading network. Positive correlations with RT indicate increasing activity for items with slow responses, or decreasing activity for fast items. As shown in **Figure 2** and **Table 3**, slow items were associated with increased BOLD signal (positive correlation) in bilateral regions including insula, IFG, IPS, and FFG, as well as in left regions at middle occipital gyrus (MOG), IPS, MFG, and pre/post-central gyrus. These findings are consistent with RT-related regions in English word naming (Binder et al., 2005; Graves et al., 2010). In addition, slow items were associated with decreased activity (negative correlation) in left MFG, right anterior MTG, and bilateral areas of precuneus, anterior cingulate cortex (ACC) and AG.

**Table 3:** Brain regions correlated to reaction time (RT).

Region	H	BA	Volume	*t*	*x*	*Y*	*z*
**Positive**							
Med. frontal G.	B	6	1583	15.77	-5	13	53
Mid./Inf. frontal G.	L	13	1685	13.19	-29	32	10
Precentral G.	L	2	255	11.67	-31	-31	66
Mid. frontal G.	L	6	250	10.01	-25	-10	52
Inf. frontal G.	R	6	281	5.88	37	12	27
Insula	R	13	533	14.05	33	28	4
Post-central G.	L	40	79	7.09	-48	-31	62
	L	40	56	6.63	-52	-36	37
Precuneus	L	7/39	444	11.32	-27	-62	34
	R	31/18	447	8.67	29	-66	25
Cuneus	L	19/18	498	11.15	-15	-67	1
	R	18	453	9.58	17	-72	5
Intraparietal sulcus	L	40	220	10.35	-41	-48	37
Mid. occipital G.	L	19	142	8.31	-33	-88	15
	L	19/18	77	6.34	-29	-80	2
Lingual G.	L	19	76	6.95	-19	-48	-3
Fusiform G.	L	37	206	6.74	-41	-63	-8
	R	37	61	10.19	43	-56	-15
Cingulate G.	B	24	73	6.99	1	4	31
**Negative**							
Cingulate G.	B	31	1341	-11.52	-1	-64	27
	B	31	127	-13.00	1	-28	40
Anterior cingulate	B	32	138	-7.21	1	44	7
Angular G.	L	22	498	-8.28	-46	-54	19
Supramarginal G.	R	39/40	71	-7.14	56	-54	32
Sup. temporal G.	R	21	54	-6.35	64	-23	-11
Mid. temporal G.	R	38	109	-8.54	52	15	-28
	R	21/22	101	-5.46	58	-15	-13
Mid. frontal G.	L	8	186	-6.82	-27	24	47
Med. frontal G.	L	10	146	-5.79	-11	55	3
Sup. frontal G.	L	6	94	-9.07	-19	31	56
	L	8	59	-5.03	-15	42	46
	L	10	50	-5.34	-21	63	3
	R	8/9	136	-8.38	17	49	35
	R	9	62	-6.74	11	64	30

*H = left (L), right (R), or both (B) hemisphere; BA, Broadmann’s area; Volume is given in number of voxels (2 mm × 2 mm × 2 mm); Coordinates (*x, y, and z*) for the peak active voxel in each cluster are given with reference to the MNI atlas. For brain regions: Sup., superior; Mid., middle; Inf., inferior; Med., medial; G., gyrus.*

Positive correlations for frequency indicated the increasing BOLD signal intensity for high frequency characters. As shown in **Figure 2** and **Table 4**, these regions included clusters along the midline in bilateral precuneus and ACC, and bilateral clusters in AG, supramarginal gyrus (SMG), and MTG. Negative correlations for word frequency were mainly found in bilateral portions of insula and MFG, and in portions of left-lateralized precentral gyrus, FFG, and precuneus.

**Table 4:** Brain regions showing significant correlations with BOLD signal for stimuli properties: word frequency, consistency, and the number of strokes.

Region	H	BA	Volume	*t*	*x*	*y*	*z*
**Word frequency**							
Positive						
Precuneus	B	7	1266	9.45	1	-69	38
Angular G.	L	39	94	13.33	-35	-81	28
	R	39	154	7.38	39	-75	29
Supramarginal G.	L	39/40	293	7.30	-54	-54	28
	R	40	475	9.27	58	-52	32
Inf. parietal lobe	L	39	83	7.15	-46	-67	44
	R	39	71	5.25	50	-63	36
Mid. temporal G.	L	21	66	5.79	-60	-34	-4
	R	21	110	4.77	60	-34	-7
Sup. temporal G.	R	13	83	7.67	41	10	-18
Anterior cingulate	B	25/24	230	9.81	1	22	-4
Precentral G.	R	6	71	6.12	58	3	9
Sup. frontal G.	R	10	54	5.80	9	61	1
Negative						
Mid. frontal G.	L	6/9	442	-10.91	-39	6	31
	R	9	62	-7.44	43	18	27
Insula	L	13	208	-8.80	-29	30	8
	R	13	86	-4.70	33	28	4
Cuneus	L	19/18	203	-6.21	-15	-67	1
Precuneus	L	7	162	-7.31	-23	-69	36
Fusiform G.	L	37	129	-7.57	-41	-50	-12
	R	37	51	-4.79	54	-50	-15
Mid. temporal G.	L	21	118	-6.67	-37	-2	-36
Sup. temporal G.	L	38	50	-4.49	-23	16	-49
Cingulate G.	L	6/32	351	-11.02	-7	15	49
Posterior cingulate	R	19	59	-5.99	23	-61	-1
	R	30	57	-5.65	9	-68	10
**Consistency degree**							
Negative						
Inf. frontal G.	L	45	515	-6.90	-41	32	2
	L	46	73	-6.69	-52	27	23
	R	45	82	-8.32	29	30	-1
	R	9	61	-5.21	48	12	27
**Number of strokes**							
Positive						
Parahippocampal G.	R	36/35	113	7.84	31	-20	-27
	L	36	51	5.17	-31	-20	-27
Calcarine sulcus	R	18	100	7.82	15	-70	7
Anterior cingulate	B	25	50	16.44	1	10	-4
Inf. occipital G.	L	18	91	6.90	-27	-86	-3
Declive	L	37	52	6.93	-31	-60	-18
Sup. temporal G.	L	38	51	6.71	-21	9	-40
Negative						
Precuneus	B	31/23	118	-8.26	1	-66	20
Angular G.	L	39	79	-6.47	-41	-70	27
	L	19	59	-6.54	-39	-79	31
	R	19	51	-4.43	54	-64	18

*H = left (L), right (R), or both (B) hemisphere; BA, Broadmann’s area; Volume is given in number of voxels (2 mm × 2 mm × 2 mm); Coordinates (*x, y, and z*) for the peak active voxel in each cluster are given with reference to the MNI atlas. For brain regions: Sup., superior; Mid., middle; Inf., inferior; Med., medial; G., gyrus.*

Correlations with consistency degree (ratio of homophones in the same phonetic family) were negative, including bilateral IFG and insula, indicating increasing neural activity in those regions for characters with more enemies than friends (See **Table 4**). This is consistent with prior studies of English word reading (Graves et al., 2010).

Positive correlations with the number of strokes (See **Table 4**) mainly occurred in right calcarine sulcus for processing of additional visual input, as well as bilateral parahippocampal gyrus, anterior and posterior cingulate. Negative correlations were in bilateral AG and precuneus, indicating more deactivation for more complex visual stimuli.

### ROIs Response for Sublexical Properties

**FIGURE 3:**
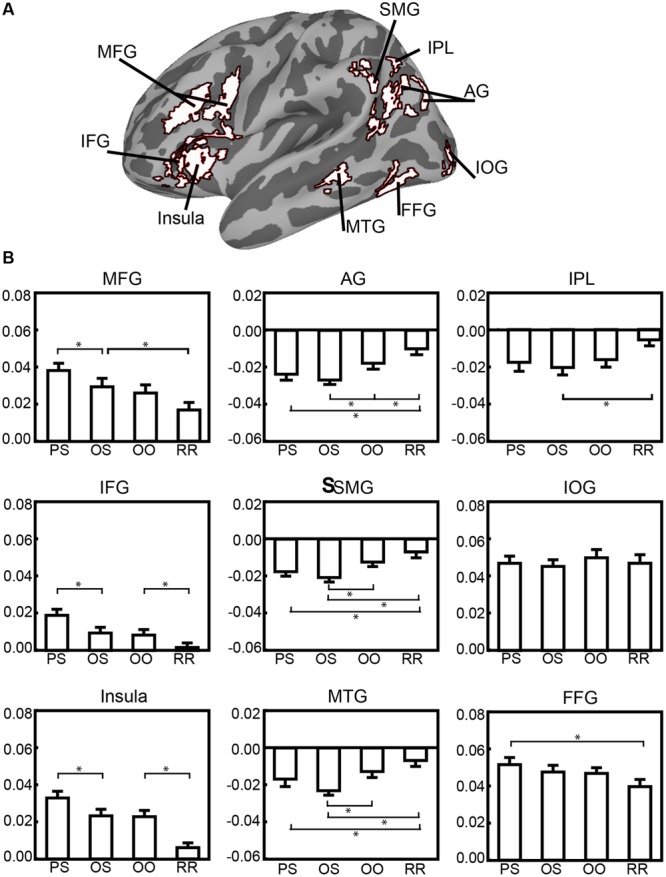
**Map of nine ROIs **(A)** identified from correlation analysis, with bar graphs **(B)** illustrating patterns of percent signal change for four types of pseudo-characters**.

In order to further examine the function of brain regions in the reading network identified both from GLM and multi-parametric approach, we conducted further analyses of ROIs identified in those analyses. Nine ROIs were selected to compare activation levels among four types of pseudo-characters (**Figure 3**). ANOVAs revealed a significant main effect of the types of pseudo-character for those ROIs (except for the inferior occipital gyrus IOG, *F*_(3,45)_ = 1.70, *p* = 0.18), indicating that although these regions were selected via a lexicality contrast, they are also modulated by the sublexical information.

*Post hoc* comparisons were conducted among four types of pseudo-characters in each ROI. The results are presented in **Table 5**. Based on contrasts designed to reveal phonological (PS > OS), semantic (OS > OO), and orthographic (OO > RR) processing, three distinct activation patterns emerged so that these ROIs were summarized as three groups engaging in O-P, O-S, and orthographic processing, respectively. The first group of ROIs located in frontal cortex (IFG and MFG) and insula. These ROIs showed a monotonic effect of sublexical information (insula, *F*_(3,45)_ = 27.82, *p* < 0.01; IFG, *F*_(3,45)_ = 17.46, *p* < 0.01; and MFG, *F*_(3,45)_ = 17.07, *p* < 0.01), in which the PS (Phonology plus Semantics) condition evoked the strongest activities and the RR (Reversed Radical) condition evoked the weakest. Both for IFG and insula, their activities were stronger for PS than for OS, and stronger for OO than for RR, indicating that they are sensitive to O-P processing. However, for MFG, its activity was incremental from RR to PS and only OS > RR and PS > OS reached significant.

**Table 5:** Paired *t*-test among four types pseudo-characters for ROIs.

Regions	PS > OS	PS > OO	PS > RR	OS > OO	OS > RR	OO > RR
**ROIs from real characters**					
Precentral, MFG	3.92^∗^ (0.001)	5.72^∗^ (0.000)	5.85^∗^ (0.000)	1.23 (0.239)	3.59^∗^ (0.003)	2.63 (0.019)
Insula	3.59^∗^ (0.003)	3.73^∗^ (0.002)	7.99^∗^ (0.000)	0.19 (0.851)	4.93^∗^ (0.000)	5.37^∗^ (0.000)
IFG	4.11^∗^ (0.000)	5.15^∗^ (0.000)	6.17^∗^ (0.000)	0.56 (0.581)	2.60 (0.020)	3.11^∗^ (0.007)
MTG	1.63 (0.125)	-1.20 (0.250)	-2.95^∗^ (0.009)	-3.43^∗^ (0.004)	-4.24^∗^ (0.001)	-2.15 (0.048)
SMG	1.65 (0.120)	-1.80 (0.09)	-4.84^∗^ (0.000)	-4.48^∗^ (0.000)	-6.05^∗^ (0.000)	-1.83 (0.087)
AG	1.31 (0.21)	-2.03 (0.06)	-4.79^∗^ (0.000)	-3.84^∗^ (0.002)	-6.83^∗^ (0.000)	-3.18^∗^ (0.006)
IPL	0.53 (0.605)	-0.28 (0.780)	-2.68 (0.017)	-0.84 (0.412)	-4.14^∗^ (0.001)	-2.49 (0.025)
IOG	0.98 (0.342)	-1.44 (0.171)	-0.01 (0.998)	-2.31 (0.035)	-0.63 (0.540)	1.60 (0.131)
FFG	1.99 (0.064)	2.24 (0.040)	3.10^∗^ (0.007)	0.34 (0.738)	2.62 (0.019)	2.25 (0.040)
**Sub-regions from conjunction**					
Common	3.47^∗^ (0.003)	3.81^∗^ (0.002)	7.14^∗^ (0.000)	-0.01 (0.995)	4.02^∗^ (0.001)	5.20^∗^ (0.000)
Frequency	3.59^∗^ (0.003)	3.43^∗^ (0.004)	7.80^∗^ (0.000)	0.30 (0.771)	5.25^∗^ (0.000)	5.21^∗^ (0.000)
Consistency	4.11^∗^ (0.001)	5.29^∗^ (0.000)	5.70^∗^ (0.000)	0.67 (0.513)	2.07 (0.060)	2.26 (0.040)

*p-values in parentheses are uncorrected; asterisk indicates significance at Bonferroni corrected *p* < 0.05 with uncorrected *p* < 0.0083 (0.05/6).*

The second group of ROIs located at MTG and temporoparietal junction (SMG and AG). All these ROIs showed similar stimuli effects (MTG, *F*_(3,45)_ = 8.12, *p* < 0.01; SMG, *F*_(3,45)_ = 12.81, *p* < 0.01; AG, *F*_(3,45)_ = 16.44, *p* < 0.01). Their activities were stronger for OS > OO, but not for PS > OS, indicating that they are sensitive to sublexical semantic processing but not to phonological processing. Only in AG, a significantly stronger effect was found for OO > RR, indicating a role in O-S processing.

The third group ROIs was related to orthographic processing (FFG and IPL). There was a significant stimuli effect both for FFG (*F*_(3,45)_ = 6.23, *p* < 0.01) and for IPL (*F*_(3,45)_ = 3.92, *p* < 0.05). Only a significant difference was observed between the strongest and weakest activated condition (FFG, PS > RR; IPL, OS > RR). No significant difference was observed for other pairs of conditions. It indicated FFG and IPL were not sensitive to O-P or O-S processing.

### Sub-Regions in Ventral Inferior Frontal Cortex and Insula

**FIGURE 4:**
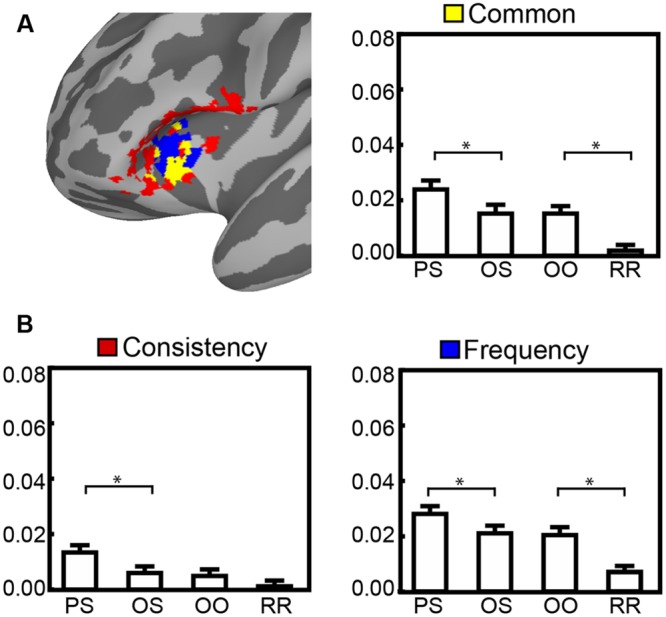
**Brain map **(A)** of conjunction analysis on inferior frontal cortex correlated to frequency (blue), consistency (red) and both (yellow), with the bar graphs **(B)** illustrating patterns of percent signal change for four types of pseudo-characters**.

From ROI analysis, we observed a similar pattern at IFG and insula. It might result from the activity of overlapping voxels drawn from these two adjacent ROIs. The ROI labeled as IFG for consistency shared some voxels with the ROI labeled as insula for frequency. To test the function of sub-regions, we created a conjunction map (shown in **Figure 4A**) by selecting voxels specific for the consistency degree, specific for the frequency and common for both. As shown in **Figure 4B**, ROI analysis revealed two types of pattern among pseudo-characters in three sub-regions, multiple comparison results were listed in **Table 5**.

For common and frequency specific sub-regions in insula, we observed a similar stimulus effect, in which the BOLD intensity was greater for PS > OS and OO > RR indicating a function of O-P processing. The difference between OS and OO condition did not reach significant indicating this region was not sensitive to O-S processing.

As for the sub-region specific for consistency degree at IFG, we observed a different stimulus effect. The PS condition evoked higher activation than three other conditions, and there was no difference among OS, OO, and RR conditions. This pattern indicated left IFG was only sensitive to phonological processing.

## Discussion

The main goal of the current study was to investigate the neural networks involved in Chinese character reading and the independent contribution of orthographic, phonological, and semantic processing. Consistent with prior studies of reading in alphabetic languages, we identified two neural pathways in the left hemisphere. One pathway relied on left posterior middle temporal gyrus (pMTG) and AG for semantic processing, and the other pathway relied on left IFG and insula for phonological processing. The cooperative division of labor of these two neural pathways implicates the neural basis for processing different types of characters. The findings are consistent with reading models that assume the same general architecture for logographic and alphabetic scripts.

### Neural Correlates for Semantic Processing

A significant finding of the present study is the neural basis of semantic processing in Chinese character reading. Since the mapping from orthography to meaning is arbitrary in alphabetic words, the logographic properties of the Chinese writing system provide an opportunity to directly examine the neural basis of semantic processing. Pseudo-characters with semantic cues were associated with stronger activity in left MTG and AG. This is potentially related to lexico-semantic processing, as semantic cues in Chinese character provide probabilistic information about the whole character. Our result is consistent with previous findings that these regions were active more for semantic tasks than phonological tasks (Price et al., 1997; Booth et al., 2006), and high, compared to low imageability words (Frost et al., 2005).

But, the engagement of left AG might go beyond the lexico-semantic processing. In current study, the left AG was not only active for lexico-semantic (OS > OO) processing, but also for orthographic (OO > RR) processing (see **Figure 3**). The result indicated a function of AG for mapping from orthography to semantics. Prior studies have shown that AG plays a role in complex information integration and knowledge retrieval (Binder et al., 2009; Bonner et al., 2013). For Chinese O-S mapping, we might suggest that AG plays a role in combining the perceptual information, integrating the meaning, and manipulating the relevant information in the semantic system (Seghier, 2013; Price et al., 2015).

Thus, the neural basis of semantic processing in reading Chinese characters relied on the involvement of left MTG and AG. The MTG associated more strongly with lexico-semantic processing, whereas, the AG associated with integration of semantic cues and other sources of information.

### Neural Correlates for Phonological Processing

Another interesting finding is the neural correlates of the phonological processing in Chinese character reading. We found a correlation with consistency effects and selectivity for pseudo-characters including phonological information in left IFG and insula. This finding is consistent with previous studies that these two regions are sensitive to consistency in Chinese (Lee et al., 2004, 2010). It is also consistent with findings in alphabetic languages (e.g., Fiez et al., 1999; Graves et al., 2010) indicating left IFG and insula could be involved in phonological processing across languages. The IFG and insula can be understood as contributing to phonological processing in the context of multiple different models of word recognition. As Taylor et al. (2013) point out in a meta-analysis of FMRI research on reading in alphabetic languages, the IFG is more strongly activated for both pseudowords and irregular words. This is consistent with the “triangle” model, in which computing phonology for both pseudowords and words with inconsistent spelling-to-sound correspondences generates a high degree of ambiguity in phonological representations. Similarly, Taylor et al. (2013) assert that in a dual-route framework, the IFG may be thought of as a phonological buffer, where ambiguities arising during grapheme-to-phoneme conversion (e.g., Rastle and Coltheart, 1999) or from computation between addressed and assembled phonological representations may play out.

The role of the insula in visual word recognition is less well-understood, although, it is engaged in visual word reading both in English (Fiebach et al., 2002) and in Chinese (Kuo et al., 2004), and is proposed to be sensitive to phonological processing (particularly sublexical spelling-sound translation, Borowsky et al., 2006). In addition, some researchers have suggested that insula is responsive to cognitive processing load (Zaccarella and Friederici, 2015) because of its increasing activity with the increasing processing demands (Bahlmann et al., 2008). This explanation is consistent with the view of insula as a function of general processing demand in English word reading (Graves et al., 2010). Consistent with this view, we found activity in the insula was strongly correlated with RT, and, further, showed effects of both phonological (PS > OS) and orthographic (OO > RR, see **Figure 3**) structure in the pseudo-characters.

### Lack of Phonological Effects in Posterior Superior Temporal Cortex

The left pSTG is a core region for sublexical phonological processing in alphabetic languages (Paulesu et al., 2000; Pugh et al., 2000), but was not found to be engaged in Chinese phonological processing in our study. A common explanation is that the pSTG is specific to alphabetic languages, and plays a specific role in “assembled phonology” (e.g., Paulesu et al., 2000; Tan et al., 2005). Chinese orthography is extremely opaque, and in some models, is assumed to lack assembled phonological processes entirely (Coltheart et al., 2001; Perfetti et al., 2005). The finding is compatible with the relatively weak (or null) activation in previous Chinese fMRI studies (Tan et al., 2005; Liu et al., 2007). This is also consistent with the finding that a patient with a lesion in the left STG showed phonological dyslexia in English but intact reading in Japanese (Wydell and Kondo, 2015). On the other hand, another possibility is that the LD task itself puts minimal demands on O-P processing, and may not be optimal for observing activity in pSTG. We have observed activity in this region for Chinese reading under naturalistic contexts (Wang et al., 2015), and in a working memory task (Wang et al., 2011; Yang et al., 2011). Furthermore, Rueckl et al. (2015) recently reported activity in this area for Chinese during a semantic decision task, that did not differ from three alphabetic languages (Spanish, English, and Hebrew).

### Middle Frontal Gyrus and Meta-linguistic Processing Demands

It is also worth mentioning the engagement of left MFG in Chinese character reading. Although, this region was proposed as a typical Chinese-specific region for reading (Siok et al., 2004), its function was a long-debated issue in prior studies. The left MFG has been linked to the semantic access (Tan et al., 2000; Luke et al., 2002), addressed phonology (Tan et al., 2005), or integrating the visual spatial analysis and the semantic (or phonological) analysis (Tan et al., 2001b). In our data, left MFG was highly correlated with frequency, but not consistency. Its function seems unrelated to phonological processing for Chinese characters, but it was sensitive to sublexical phonological (PS > OS) processing for pseudo-characters. These results reflect that the processing demand drove the activity of the left MFG. This notion is in concordance with the view that left MFG was linked to working memory (Liu et al., 2007) for retaining the character form until both meaning and pronunciation was retrieved. It hence can explain the observation that left MFG was active in alphabetic word processing under particular task demands (Nakamura et al., 2012), and in naturalistic reading (Wang et al., 2015).

### The Interaction between Semantic and Phonological Processing

In addition to patterns of activity that were apparently specific to O-P and O-S processing, we also found evidence for interactions between semantic and phonological processing. For example, areas supporting phonological processing (IFG, insula) showed a gradient activation from RR to PS pseudo-characters. Meanwhile, areas supporting semantic processing (MTG, AG) were deactivated gradually from RR to PS. A similar trade-off effect has been found in English word reading (Frost et al., 2005), and has been interpreted in terms of the connectionist triangle model (Harm and Seidenberg, 2004) of reading (Carreiras et al., 2014).

Consistent with our findings, some patient studies have shown interactions between semantic and phonological processing in reading logographic characters. Bi et al. (2007) presented a case study of a patient with semantic deficits following a left temporal lobe ischemic damage, whose reading performance depended on whether he knew the meaning of the target character: he could correctly name those characters for which he could also provide a definition, whereas, for those characters he could not understand, his reading performance was poor overall. Nonetheless, for characters he could not understand, his performance was better for regular-consistent than irregular-inconsistent characters, indicating that phonological processing was engaged while the semantic processing was unavailable. This interaction is consistent with findings in neuropsychological studies both in alphabetic (Woollams, 2014) and logographic scripts (e.g., see review of Chinese, Weekes et al., 2006; review of Japanese, Sato, 2015), in which damage to visual/orthographic, phonological, and semantic processing abilities are the root causes of different types of acquired dyslexia. This is consistent with connectionist approaches to understanding reading in general, and with our own model of reading Chinese characters (Yang et al., 2009, 2013).

The current study is the first application of a multi-parametric approach in investigating the neural network for Chinese character reading. The findings were consistent with studies in alphabetic word reading using the same approach (Graves et al., 2010). One difficulty of the multi-parametric approach is to avoid the intercorrelation among variables. Our analysis used data from a previous study (Yang et al., 2012) that manipulated stimulus properties relevant to the differential contribution of orthographic, phonological, and semantic properties to character recognition, so that phonological consistency was not correlated with other variables. However, frequency was significantly correlated with number of strokes and, unavoidably in a LD experiment, with RT. Further study using multi-parametric approach should deliberately select stimuli to avoid intercorrelation among variables, and include more semantic factors such as imageability.

## Conclusion

In this work, we sought to determine the cortical substrate of the relationship between orthography-to-phonology and orthography-to-semantics processing in visual word reading. We found a shared neural network both for real and pseudo-characters, and evidence for cooperative division of labor between semantic and phonological processing. The finding provided neural evidence for the connectionist model that assumes a general framework for reading both alphabetic words (Seidenberg, 2011; Carreiras et al., 2014) and Chinese characters (Yang et al., 2009, 2013).

## Author Contributions

XW, JZ, and JY designed research; XW, RZ, and JY analyzed data; XW and JY interpreted data; XW, JZ, and JY wrote the paper.

## Conflict of Interest Statement

The authors declare that the research was conducted in the absence of any commercial or financial relationships that could be construed as a potential conflict of interest.
